# An opinion evolution model for online social networks considering higher-order interactions

**DOI:** 10.1371/journal.pone.0321718

**Published:** 2025-04-16

**Authors:** Quan Liu, Yuekang Yao, Meimei Jia, Huizong Li, Qiru Pan

**Affiliations:** 1 School of Artificial Intelligence and Software Engineering, Nanyang Normal University, Nanyang, China; 2 Henan Provincial Engineering Research Center for Image Big Data Intelligent Processing, Nanyang, China; Université de Bourgogne: Universite de Bourgogne, FRANCE

## Abstract

As the number of users in online social networks increases, the diffusion of information and users’ opinions on events become more complex, making it difficult for traditional complex networks to accurately capture their characteristics and patterns. To address this, this paper proposes an online social network opinion evolution model that accounts for higher-order interactions. The model incorporates the higher-order effects of group interactions and introduces the acceptance, non-commitment, and rejection dimensions from social judgment theory. Different approaches, such as acceptance, neutrality, and contrastive rejection, are adopted when individuals exchange opinions with their neighbors. Through numerical simulations, it is shown that higher-order interactions significantly enhance the speed and coverage of information propagation. When the interaction dimensions are appropriate, increasing the average size of hyperedges significantly contributes to the formation of consensus. In contrast, simply increasing the number of hyperedges that nodes are involved in has a limited impact on consensus formation. This work provides a theoretical and model-based foundation for better understanding the dynamics of opinion evolution in social networks.

## 1 Introduction

With the development of the digital landscape, social media platforms have become deeply embedded in every aspect of daily life, evolving into a primary channel for information dissemination. Platforms such as TikTok, Twitter, Facebook, and Weibo, with their ease of use, broad content coverage, and low entry barriers, have attracted large numbers of users by disseminating information primarily through short posts and videos. At the same time, negative and misleading voices can easily spread on these platforms, which can have a significant impact on public sentiment [1,2].Understanding how information spreads and how public attitudes evolve is crucial. In previous studies, much attention has been focused on examining the information dissemination patterns and mechanisms within online social networks. In traditional complex networks, it is assumed that individuals form their opinions through pairwise interactions between nodes. Scholars have developed propagation models based on the characteristics of information contagion [3–5]. However, the formation of individual opinions takes place within a broad social network and group interactions, which significantly influence the shaping and evolution of these opinions. Graph structures can only model pairwise relationships between entities, and this approach fails to capture the higher-order responses involved in opinion formation and diffusion within real-world groups [6–8].

Existing studies represent these complex higher-order associations using hypergraphs, where a hyperedge can connect an arbitrary number of nodes. As a result, hypergraphs are naturally suited for modeling complex systems. Higher-order associations are also common in the real world. Such as Facebook groups, Twitter communities, LINE groups, and WeChat group chats. Gong [9] uses hyperedges in hypergraphs to represent community relationships between users and develops an online social hypernetwork model for information dissemination.Simulation results in [10] show that hypergraph-based network structures closely match the structure of virtual communities.In [11], hypergraphs are used to link news, users, and events within a unified model for rumor detection.The study in Zeng [12] combines hypergraph structures and large language models (LLMs), analyzing the relationships between nodes (users) and higher-order structures (such as co-participated discussion groups or communities) to explore the link between personality traits and social behaviors. In multilayered hypernetworks, hyperedges can connect to other hyperedges, forming a hierarchical structure, which in social networks manifests as relationships between different interest groups [13,14].

In existing hypergraph studies, the diffusion of information flow can reach the whole hypergraph through a hyperedge, thus revealing the characteristics of group propagation [15]. Additionally, nodes in a hypergraph can influence multiple hyperedges, helping identify individuals with significant roles across multiple groups, such as key nodes in information spread within various social circles [16]. Literature [17] introduced a new hypergraph model that incorporates community structure and opinion divergence. In epidemic modeling, Arruda et al. [18] studied social contagion dynamics on hypergraphs, providing insights for modeling higher-order structural dynamics. Higham et al. [19] explored extinction thresholds in epidemic models on hypergraphs and proposed a new disease control strategy based on hypergraph structures. John et al. [20] studied the extinction phenomena of diseases in SIS models on dynamic graphs and hypergraphs. Antelmi et al. [21] proposed a time-varying hypergraph design method for studying and predicting dynamic epidemic spread patterns. Gong et al. [22] combined hypernetwork models with the SEIR model to create an online social hypernetwork information diffusion model based on user and information attributes. Rohit et al. [23] investigated the nonlinear dynamics of consensus formation on hypergraphs, allowing interactions within hyperedges of arbitrary cardinalities. Xu et al. [24] extended the classical Friedkin-Johnsen model from graphs to hypergraphs to capture common higher-order interactions in real networks, particularly in social networks. Hendrik et al. [25] analyzed opinion changes in group discussions and found that, compared to two-person discussions, consensus formation in group discussions is more gradual and less prone to abrupt agreement or breakdowns. Nicholas et al. [26] further explored information and disease propagation in networks, finding that group interactions significantly influence the spread, and their model incorporating links and triangles reveals how network structure heterogeneity mitigates abrupt changes in the propagation process. James et al. [27] focused on group discussions on hypergraphs, particularly the opinion evolution within three-person groups, and predicted the trends in opinion change through mathematical models. Tan et al. [28] found that higher-order interactions make rumor propagation more complex, potentially leading to bistability and discontinuous transitions. Their study also explored intervention strategies for improving public media literacy and building spaces for truth, concluding that these measures can effectively reduce the scale and speed of rumor spread, especially when implemented collaboratively.

In the field of opinion evolution, researchers have primarily focused on improving interaction models. For example, the Hegselmann-Krause model and the Deffuant-Weisbuch model, with most simulations conducted on complex networks. Kan et al. [29] proposed an adaptive DW model that adjusts the trust boundaries between individuals, allowing the model to better simulate the process of group opinion formation in real-life scenarios. Yun et al. [30] introduced an improved DW model that incorporates both explicit and implicit opinions of individuals within a social network. Ling et al. [31] proposed an enhanced HK model to facilitate consensus formation in group decision-making, incorporating interval-valued fuzzy preference relations and confidence levels. Building upon this, we find that social judgment theory offers a new perspective for opinion research. When updating their views, individuals tend to choose communication partners based on the principle of similarity, preferring to interact with neighbors holding similar opinions [32,33]. During opinion interactions, understanding the attitudinal dimensions of the target audience can help develop more effective communication strategies. When attempting to change someone’s attitude, information should fall within their acceptance or non-issue zones, avoiding direct conflict with their rejection zone.

These studies have expanded research in information diffusion and opinion dynamics. Most of the research sets the change of user opinions within a theoretically infinite time frame, typically ending the simulation once a stable state is reached. However, this approach does not accurately reflect the dynamic changes of public opinion in online social networks. In online social networks, not all individuals actively participate in discussions about a particular event; some may only be recipients of information rather than its disseminators. However, there is limited research that combines information diffusion with opinion evolution in online social networks, considering higher-order opinion interactions between multiple nodes. This paper uses hypergraphs to represent higher-order interactions between nodes, incorporates the information diffusion process, and improves the H-K model by integrating social judgment theory, thereby constructing a hypergraph-based model for opinion evolution in online social networks that accounts for higher-order interactions. This study simulates the higher-order interaction process between multiple nodes in online social networks, revealing how opinions spread within these networks and how individuals receive, process, and update their views in this process. This simulation not only considers the dynamics of information diffusion but also reflects individual interaction patterns and the selectivity of information reception in social networks, with extensive experiments validating the effectiveness of our model. The model in this paper aims to reveal how structural factors influence opinion convergence or divergence, rather than advocating for any specific political or policy orientation.

## 2 Theoretical background

### 2.1 Relevant concepts of hypergraphs

Hypergraphs connect multiple nodes through hyperedges, effectively representing multi-party relationships and complex interactions within a system. Unlike simple graphs, which can only represent relationships between two nodes, hypergraphs have a higher dimensionality, allowing them to capture richer structural information [33]. In social networks, nodes represent users, and hyperedges represent the social relationships in which users participate. For example, multiple employees of a company may be connected through the same hyperedge, reflecting the diversity of group interactions. In weighted hypernetworks, the weight of a hyperedge can reflect its importance or frequency, making the model more accurate in representing real-world scenarios. Hypergraphs not only extend the concept of traditional networks but also excel in handling tasks involving multi-party interactions. They are widely used in fields such as social network analysis, knowledge graph construction, and biological network analysis, providing a powerful tool for the study of complex systems.

The mathematical definition of a hypergraph [34] is as follows: Let the set of vertices $V=\{v_{1},v_{2},v_{3},...,v_{n}\}$ be a finite set, and the set of hyperedges $E=\{e_{1},e_{2},e_{3},...,e_{n}\}$be a finite set. The hypergraph *H=(V,E)* is defined as the collection of hyperedges, where the hyperedge$e_{i}=\{v_{i_{1}},v_{i_{2}},v_{i_{3}},...,v_{i_{n}}\},\;(i=1,2,3...,m,v_{i_{j}}\in V)$. If two nodes are neighbors (adjacent), they belong to the same hyperedge. If the number of nodes contained in each hyperedge in a hypergraph is exactly *k*, then such a hypergraph is said to be a consistent hypergraph of order *K*, i.e., a uniform hypergraph. If *k*=2, the hypergraph degenerates into a regular graph.

The key advantage of hypergraphs lies in their ability to directly model higher-order interactions between multiple nodes, going beyond the binary interactions typically found in traditional networks.This modeling approach naturally captures and represents higher-order motifs, making one of the important functions of hypergraph methods the accurate representation of these motifs’ statistical properties—properties that are often difficult to directly reflect in traditional network models, such as the BA model [35,36].Even if regular network and hypergraphs theoretically possess the same higher-order motif statistical properties, their dynamic behaviors (such as information propagation, synchronization, community detection, etc.) may still exhibit significant differences.This is primarily because hypergraphs can directly model and reflect the global effects of higher-order interactions, while regular network can only indirectly represent these effects through local binary relationships [37].

### 2.2 Social judgment theory

Social judgment theory involves how people process information, form attitudes, and respond to differing opinions. Sherif & Hovland [38] proposed social judgment theory in 1961, asserting that everyone holds a basic stance on a particular issue, referred to as the “anchor point. “The anchor point reflects an individual’s core attitude or opinion on an issue, serving as the benchmark for judging other opinions. Based on the anchor point, each individual has an opinion dimension for a particular issue, consisting of three main ranges:

(1) Acceptance range: The range of opinions or attitudes that an individual can accept or agree with.
(2) Non-commitment range: The range of opinions or attitudes that an individual feels neutral or indifferent toward.
(3) Rejection range: The range of opinions or attitudes that an individual cannot accept and will oppose.

**Fig 1:**
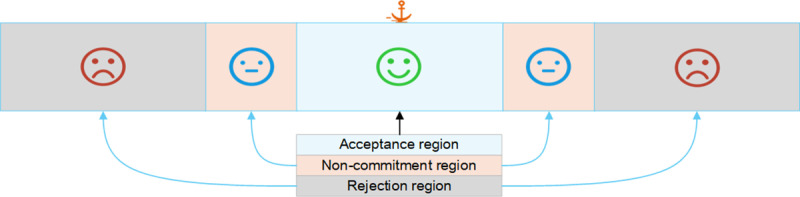
Schematic diagram of perspective dimensions. There are three areas near the opinion anchor point.

Social judgment theory predicts that attitude change is only possible when a new opinion falls within an individual’s acceptance range or non-commitment range. If the new opinion falls within the rejection range, the individual is unlikely to change their attitude and may even become more entrenched in their original position. The theory also discusses the concept of involvement, which refers to the extent of an individual’s emotional or cognitive investment in an issue [39]. A user’s level of involvement determines the extent of their acceptance, non-commitment, and rejection ranges. Highly involved users tend to have a larger rejection range and frequently encounter information that aligns with their views, making them more stubborn due to selective exposure. Users with moderate or low involvement have larger acceptance and non-commitment ranges, allowing them to accept more opinions from others and easily change their own opinions. This is similar to the HK model, where an individual combines their previous opinions with those observed from others. A schematic diagram is shown in Fig 1.

## 3 Model description

### 3.1 Structure of hypergraphs

Network topologies vary, including fully connected networks, BA scale-free networks, small-world networks, ER random networks, community structure networks, and hierarchical networks. The topology of social networks not only affects the flow and speed of information dissemination but also influences individual and collective behavior patterns, and can even shape collective cognition and culture. Based on the research in [40–43], we introduce heterogeneity to the BA scale-free hypernetwork. Since group sizes in the real world are always heterogeneous, we consider a BA non-uniform hypergraph as the foundational network.

**Fig 2:**
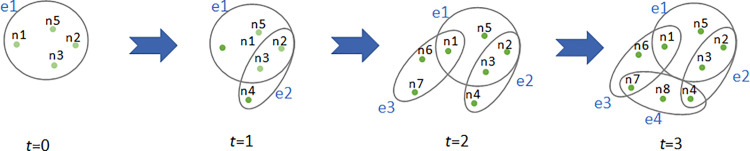
Example of hypergraph generation. t from 0 to 3, a total of 8 nodes.

Generation of the BA Structure Non-uniform Hypergraph:

(1) Initialization: Initially, the network contains a small number of nodes and a hyperedge that includes these nodes.
(2) Network Growth: In each time step, 1 to *m*_1_ new nodes are added, and a new hyperedge is formed by connecting 1 to *m*_2_ existing nodes. A total of *M* unique hyperedges are created. After *t* time steps, the network contains *M*=1+*t* hyperedges. When *m*_1_ and *m*_2_ are both equal to 1, the hypernetwork degenerates into the original pairwise interaction network.
(3) Scale-free Mechanism: New nodes are added to the network, and when forming new hyperedges with old nodes, the probability of selecting old nodes is based on their degree. Nodes with higher degrees are more likely to be chosen.A schematic of the hypergraph generation is shown in Fig 2.

### 3.2 Communication and opinion interaction strategies

Hyperedge update: A hyperedge is randomly selected in the network, which contains multiple nodes that together form an interactive set. For each node in the selected hyperedge, the values are adjusted according to predefined update rules. These rules are based on the node’s current state, historical behavior, or other relevant factors. At each time step, each node exchanges information with its neighboring nodes within the same hyperedge. This exchange is bidirectional, allowing nodes to share, integrate or coordinate their information with each other. At each time step, the above update and exchange process is repeated until the network reaches a steady state or a specific number of iterations are satisfied. The states of the nodes are categorized as S-state, E-state, I-state and R-state. The views opinions of the nodes are of continuous type between [0,1]. The initial node’s opinion *O*_*i*_ distribution is uniform, and each node has a unique latency and recovery period, sized within a few time steps. The SEIR model used in this study effectively captures both the incubation period and the dissemination process of information, particularly the intermediate phase where individuals transition from merely encountering information to actively spreading it [9,44]. Additionally, this study reduces the risk of overfitting by conducting extensive simulations, incorporating random factors, and accounting for individual differences.

**Fig 3:**
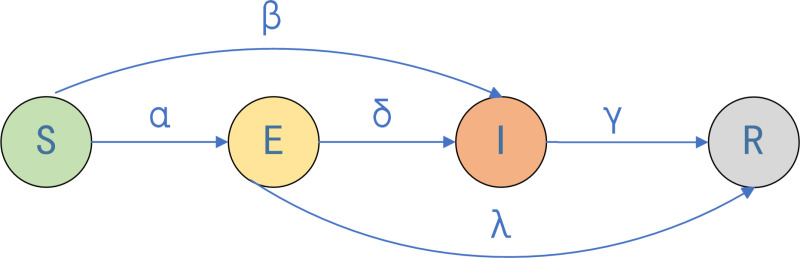
State transition diagram. *α, β, γ, δ, λ* for different conversion rates.

For each time step *t*, the nodes are updated as follows:

(1) Initially, the nodes of the entire hypernetwork are in an unknown state, and *n*_0_ I-state nodes are put into propagation.

(2) I-state nodes begin to disseminate information. S-state nodes enter into the E-state with probability *α* under the influence of neighboring nodes in the I-state, knowing the information but not disseminating it, or else enter into the I-state with probability *β* to disseminate it. E-state nodes enter the I-state with probability *δ* to disseminate it at the end of the latency period, or lose interest in the topic with probability *λ* to change to the R-state. I-state nodes lose interest at the end of the recovery period with probability *γ* to change to the R state. The R state is left unchanged. The schematic diagram is shown in Fig 3.

**Fig 4:**
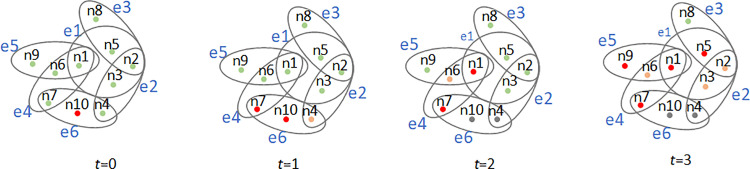
Dynamic diffusion processes of information on hypergraphs. *n*=10, *t*=3, initially only one infectious state node.


Fig 4 illustrates the dynamic diffusion of information on a hypergraph.: the green and red colors represent the S-state and I-state, respectively. At the initial time step *t*=0, one node (*n*_7_) state is randomly selected to be set to I-state, and all other nodes are in S-state. At time step *t*=1, since node *n*_7_ is in the hyper edges *e*_4_ and *e*_6_, we assume that nodes *n*_1_, *n*_4_, and *n*_10_ go into the I-state and *n*6 goes into the E-state. At time step *t* = 2, *n*_7_ and *n*_4_ return to the R-state, *n*_3_, *n*_5_, and *n*_9_ enter the E-state, and *n*_2_ enters the I-state. At time step *t*=3, n_1_, *n*_6_, and *n*_10_ return to the R state, and all the remaining nodes change to the I state.

$$\{\begin{array}{l} \frac{dS(s)}{dt}=-\alpha S(t)E(t)-\beta S(t)I(t),\\ \frac{dE(s)}{dt}=\alpha S(t)E(t)-(\delta +\lambda )E(t),\\ \frac{dI(s)}{dt}=\delta E(t)+\beta S(t)I(t)-\gamma I(t),\\ \frac{dR(s)}{dt}=\lambda E(t)+\gamma I(t). \end{array}$$ (1)

$$\{\begin{array}{l} S(t)+E(t)+I(t)+R(t)=1,\\ \alpha +\beta =1,\;\alpha >0,\beta >0,\\ \delta +\lambda =1,\delta >0,\lambda >0,\\ \gamma >0. \end{array}$$ (2)

Eq. The four sub-equations in (1) represent the rate of change of the number of nodes in the S-state, E-state, I-state, and R-state over time. Eq. The four sub-formulas in (2) represent the constraints on each parameter in the SEIR model.

(3) Node’s opinion updating process:Initially, each node has an opinion of its own and updates its opinion only after it learns the information, i.e., E-state and I-state, E-state indicates that the node discusses the information within the hyperedge after it learns the information and still hesitates whether to disseminate the information or not, at this time, E-state and I-state nodes can interact with each other with their opinions, and the opinion of the I-state nodes can be observed by the nodes within the same hyperedge, and the I-state nodes can propagate information to the hyperedge where it is located and interact with opinions.S-state and R-state nodes are in the state of unknown and removed and do not update their opinions.

In the real world, there are inevitable status differences between nodes, such as opinion leaders and ordinary users, etc., and the asymmetry of their influence is a non-negligible factor in opinion interaction. We introduce the node influence function in order to describe the influence difference between nodes. In order to facilitate the description, the use of *n*_*i*_ represents the nodes in the network, the value represents the node’s serial number, to Fig 4 any moment as an example, define the following concepts and comply with the representation.

Definition 1: The hyperdegree of a node$d_{H}(n_{i})$, represents the number of hyperedges where node *n*_*i*_ is located. For example$d_{H}(n_{i})=3$.

Definition 2: The influence weight of node j within hyperedge *e*_*i*_ is:


$$w_{ij}=\frac{d_{H}(n_{i})}{\sum_{j\in e_{i}}d_{H}(n_{j})}$$ (3)

Where $\sum_{j\in e_{i}}d_{H}(n_{j})$is the sum of degrees of all nodes within the hyperedge *e*_*i*_. Obviously, the greater the hyperdegree of node *n*_*i*_, the greater the influence weight on the remaining nodes within the hyperedge. Example:


$$w_{11}=\frac{d_{H}(n_{1})}{d_{H}(n_{1})+d_{H}(n_{2})+d_{H}(n_{3})+d_{H}(n_{5})}=0.3$$

According to social judgment theory, nodes have 3 dimensions in the vicinity of their opinion values, acceptance domain, non-commitment domain, and rejection domain.$O_{a}(i),O_{n}(i)$and$O_{r}(i)$ distributions represent acceptance, non-commitment, and rejection domains, i.e., prerequisites for node opinion interactions. Opinion updating is performed when the node opinion mean and node opinion difference within the hyperedge are in the acceptance and non-commitment domains, and opinion comparison (rebound) is performed in the rejection domain.

We quantify the opinion averages within the hyperedge as the weighted opinion summation averages for each node:


$$O_{ei}(t)=\frac{1}{\sum_{j\in e_{i}}w_{ij}}\sum_{j\in e_{i}}w_{ij}O_{j}(t)$$ (4)

The acceptance region $O_{a}(i)$: $\left|O_{i}(t)-O_{ei}(t)\right|\leq \varphi_{a}$, which indicates that the nodes and the hyperedge opinions have a small difference in the mean value and can be assimilated to each other.

Assimilation rule (within the acceptance zone): assimilation occurs if the distance between the node’s opinion and the weighted opinion sum of the nodes within the hyperedge is within the acceptance zone.

The opinion of the node moves closer to the weighted opinion sum of the nodes within the hyperedge:


$$O_{i}(t+1)=O_{i}(t)+\mu (O_{ei}(t)-O_{i}(t))$$ (5)

*μ* is the convergence parameter between [0,1].

The non-commitment region $O_{n}(i)$: $\varphi_{a}\leq \left|O_{i}(t)-O_{ei}(t)\right|\leq \varphi_{r}$ which indicates that the node is in a neutral or uncertain state with a large gap between the mean value of the node and the mean value of the hyperedge’s opinion, but not to the extent of complete rejection.

Neutrality rule (within the non-commitment zone): if the distance between a node’s opinion and the weighted sum of the nodes’ opinions within the hyperedge is within the non-commitment zone, normal communication takes place.

The opinions of the nodes remain the same or are fine-tuned according to the communication:


$$O_{i}(t+1)=\eta O_{i}(t)+(1-\eta )O_{ei}(t)$$ (6)

*η* is the convergence parameter between [0,1].

The rejection region $O_{r}(i)$: $\left|O_{i}(t)-O_{ei}(t)\right|\geq \varphi_{r}$ indicates that the difference between the node and the mean value of the hyperedge opinion is very large, which leads to the occurrence of the contrast effect (rebound effect).

Contrast (bounce) rule (within the rejection zone): the contrast effect occurs if the distance between the node’s opinion and the sum of the node’s weighted opinions within the hyperedge is within the rejection zone.

The opinions of the nodes deviate in the opposite direction:


$$O_{i}(t+1)=N(O_{i}(t)-\psi (O_{ei}(t)-O_{i}(t)))$$ (7)

where ψ>0is the divergence parameter and N(x)is a normalization function that ensures that the updated opinion values are within the legitimate range.

(4) When the state of all the nodes no longer change or there is a slight change is called to reach the steady state in the process of information propagation, the final state of the node is the removed state, the nodes in the removed state do not change.

## 4 Experiments

In the next experimental phase, our main task is to evaluate the rationality and effectiveness of the model. In the experimental part of this paper, we first conduct simulation experiments on real datasets, and then make a comparative analysis with BA scale-free networks, WS small-world networks, and BA hypernetwork. We will delve deeper into the mechanism of information dissemination and the effect of network topology on the dissemination of opinions. At the same time, we will also focus on the factors in the evolutionary process and how they shape the spread of information and the evolution of opinions. To ensure the stability and reliability of the experimental results, we will conduct 50 repetitions of each experiment under uniform experimental conditions and calculate the average value. Such a strategy of repeating experiments will help reduce random errors and improve the accuracy of the results.

In this paper, we utilize the mean of all nodes’ opinions *M* and the standard deviation of opinions $\sigma_{x}$at steady state as the parameters reflecting the overall opinions.


$$M=\frac{1}{N}\sum_{i=1}^{N}O_{i}$$ (8)

$$\sigma_{x}=\sqrt{\frac{1}{N}\sum_{i=1}^{N}{\left[O_{i}(t)-\bar{O}(t)\right]}^{2}}$$ (9)

Where Eq. (8) in which *N* represents the total number of nodes, $O_{i}$represents the opinion value of node *i* at steady state, $O_{i}(t)$represents the opinion value of node *i* at moment *t*, and $\bar{O}(t)$denotes the average value of all the individual opinions at moment *t*. Eq. (9), $\sigma_{x}$represents the standard deviation of the opinions, and a larger value of $\sigma_{x}$indicates a more dispersed opinion, and the value of $\sigma_{x}$tends to zero when the public opinion is in agreement. In hypergraphs, the calculation of links is indeed different from that in regular network. This paper counts only those edges that are not contained within other hyperedges. This approach more accurately reflects the characteristics of hypergraphs, preventing bias from repeated counting [45]. In regular network, higher-order structures (such as cliques) also exist. However, these are typically treated as approximations of higher-order interactions. Even if regular network adopt a hypergraph-like edge-counting method, they cannot fully replicate the characteristics of hypergraphs. The higher-order structures in regular network are implicit, altering the network’s topological characteristics and significantly increasing computational complexity. The data for this experiment are the generated results of modeling the real network with the generated network.

### 4.1 Experiments on real datasets

In this paper, we use the hypergraph dataset of Enron e-mails to verify the validity and reasonableness of our model. This dataset reveals the core and edge structure of Enron’s communication network through hypergraph modeling [46]. Specifically, the dataset contains 4423 nodes representing different email addresses and is categorized into “core” and “edge”. Among them, the 146 core nodes are those personal email addresses that were made public in the FERC investigation. Each hyperedge represents an e-mail message consisting of a set of e-mail addresses containing at least one core node. This design ensures that the set of core nodes constitutes the hit set of the hypergraph, i.e., this subset intersects every hyperedge in the hypergraph. The largest hyperedge in the dataset contains 25 nodes, i.e., the rank of the hypergraph is 25. the entire dataset covers 15,653 such hyperedges, providing us with a wealth of information to study and analyze the network structure.

**Fig 5:**
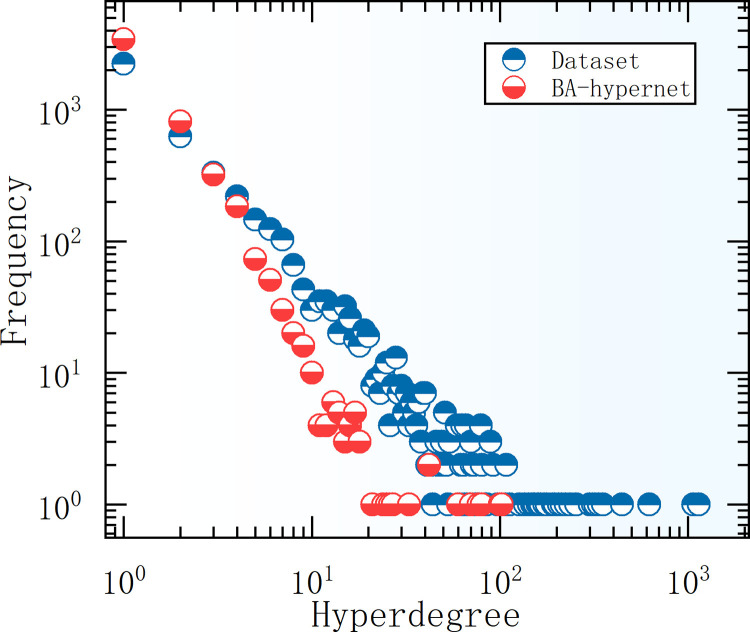
Node degree distribution diagram. The squares represent the distribution in the real dataset and the circles represent the generated dataset.

We preprocessed the dataset by removing duplicate hyperedges. The total number of hyperedges is 5734, the total number of nodes is 4423, the average node degree is 6.81, and the average hyperedge size is 5.25. We analyze its node degree distribution by comparing it with the node degree of the BA hypernetwork generated in Section 3.1, as shown in Fig 5. From Fig 5, we can find that the node hyper-degree conforms to a power law distribution.

Experimental parameter configuration: the initial opinions of all nodes obey a uniform random distribution of [0,1]. Initially, one node is randomly selected for propagation and the rest of the nodes are in unknown state. Other parameters are set as follows:


α=0.3,β=0.7,δ=0.7,λ=0.3,γ=0.3 (10)

**Fig 6:**
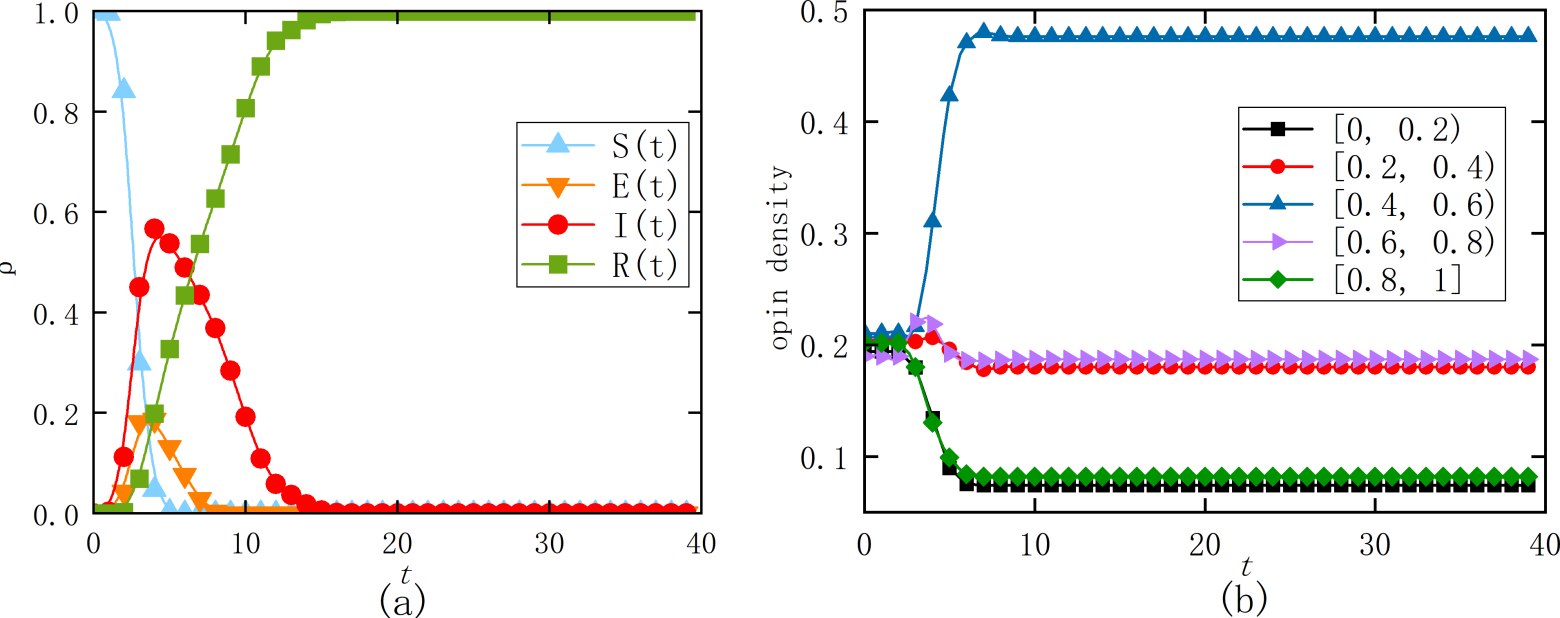
Node density curve (a) and final state opinion density distribution (b) under different states. (a) Diffusion of information flow (b) Distribution of users’ opinions evolving over time.

Fig 6(a) shows the density curves of unknown state nodes S(t), latent state nodes E(t), infected state nodes I(t), and removed state nodes R(t) over time for the real dataset. From Fig 6(a), it can be seen that as the density of infected nodes increases, the density of unknown state nodes rapidly decreases to 0. The density of latent state nodes quickly reaches a peak near 0.2. The density of infected state nodes rapidly reaches a peak of around 0.6, then gradually decreases until it stabilizes at a density of 0. After a certain period, the density of removed state nodes increases rapidly and eventually stabilizes. In combination with the hypergraph structure, the simulation analysis reveals that information propagation within a group structure is explosive. Once an infected node appears in a group chat (hyperedge), it can quickly spread information to a large number of other nodes. Within a short time, nearly all nodes in the network become aware of the information, and as the topic loses its intensity, the infected state nodes gradually transition to the removed state. Overall, this process not only demonstrates the dynamic nature of information propagation but also highlights the crucial role of the hypergraph structure in this process.

Fig 6(b) presents the density curves of opinion evolution for nodes, with acceptance range values in [0, 0.4), non-commitment range values in [0.4, 0.55), and rejection range values in [0.55, 1], over time. From Fig 6(b), it can be seen that in the initial state, the opinions of each node are uniformly distributed within [0, 1], with a density of 0.2 in each of the five intervals. As time progresses, opinions in the intervals [0, 0.2) and [0.8, 1] rapidly decrease, while the opinions in the intervals [0.2, 0.4) and [0.6, 0.8) change more gradually. Opinions in the [0.4, 0.6) interval increase sharply, with the largest change in magnitude. At steady state, the mean opinion is *M*=0.50±0.01, and the standard deviation is $\sigma_{x}$=0.19±0.02. With an initial uniform distribution of opinions$O_{i}$, extreme opinions initially dominate. As information spreads, users in the network interact and update their opinions, reducing the gap in opinions within hyperedges. The radius of opinion interaction increases, and the number and influence of extreme views gradually decrease, with most users’ opinions converging toward the average. This evolutionary pattern is similar to the dynamic processes described by the HK and DW models.

### 4.2 Analysis of the impact of network topology on opinion evolution

In this paper, four different network topologies are set up to compare the propagation and opinion evolution, namely BA networks with higher-order motif, BA scale-free network, WS small-world network and BA hypernetwork. The hypernetworks are generated according to the algorithm presented in Section 3.1, with m_1_ randomly selected within (1,5) and m_2_ randomly selected within (1,10). Table 1 provides detailed information about the different network structures. BA networks with higher-order motif are transformed by BA hypernetworks counting higher-order motif.

**Table 1:** Details of different network structures.

Dataset	*N*	*E*	Node average degree	Hyperedge average size
BA scale-free	5000	19984	7.9936	/
WS Small World	5000	21561	8.6244	/
BA hypernetwork	5000	1653	2.79	8.44
Real dataset	4424	5734	6.81	5.25
BA(higher-order motif)	5000	9996	4	/

Experimental parameter configuration: the initial opinions of all nodes obey a uniform random distribution from 0 to 1. Initially, one node is randomly selected for propagation, and the rest of the nodes are unknown states. Other parameters are set as follows:


α=0.3,β=0.7,δ=0.7,λ=0.3,γ=0.3 (11)

**Fig 7:**
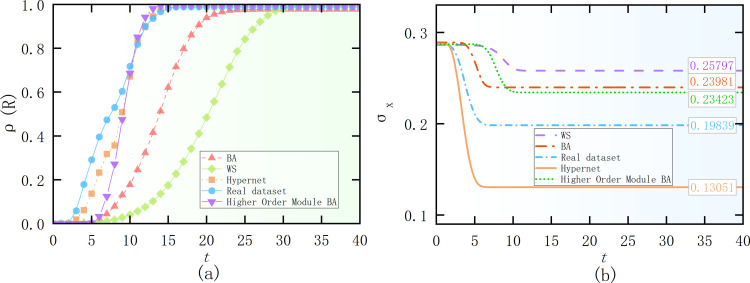
Information propagation (a) and standard deviation (b) under different topological network structures. (a) R-state node density for four network topologies (b) Standard deviation of the overall view over time for four network topologies.

Fig 7(a) presents the time evolution of the density ρ(R)of removed state nodes for different network topologies. From Fig 7(a), it is observed that each network is capable of rapidly and widely propagating information. Among them, two hypernetworks propagate information the fastest, followed by the BA networks with higher-order motif and the BA scale-free network, with the WS small-world network taking the longest time to spread. When hot information spreads through the network, hypernetworks with BA scale-free, WS small-world, and community structures allow the information to diffuse quickly along diverse paths. The presence of hyperedges enables users within a group to spread information to the entire network in a shorter time and with fewer transmission steps. Fig 7(b) shows the standard deviation results for different network topologies based on the interaction domain parameters from Section 4.1. The mean value *M* is 0.5 (±0.01) for all cases. The final standard deviation $\sigma_{x}$is larger for the BA networks with higher-order motif, the BA scale-free and WS small-world networks, indicating that user opinions are more dispersed. However, the standard deviation $\sigma_{x}$for the two hypernetworks stabilizes below 0.2, indicating that a soft consensus is approximately reached. From the model analysis: In social networks, hyperedges are typically formed based on common interests or goals, leading to the creation of highly homogeneous communities. This homogeneity means that members within a group are more likely to accept opinions and beliefs that are similar to their own. Moreover, in such networks, users are influenced not only by their directly connected friends but also by the broader community members connected through hyperedges. This multi-level social influence helps accelerate the formation of consensus, making it easier for community members to align their opinions and attitudes. Table 2 shows the difference between regular networks and hypergraphs.

**Table 2:** Differences between regular networks and hypergraphs.

Features	Hypergraph	Regular network
Interaction mode	Many-to-many group chats (hypergraph side)	One-to-single follow/forward (side)
Information dissemination efficiency	High (group effect)	Low (information dissemination delay)
Opinion evolution	hyperedge size control	central node influence

**Fig 8:**
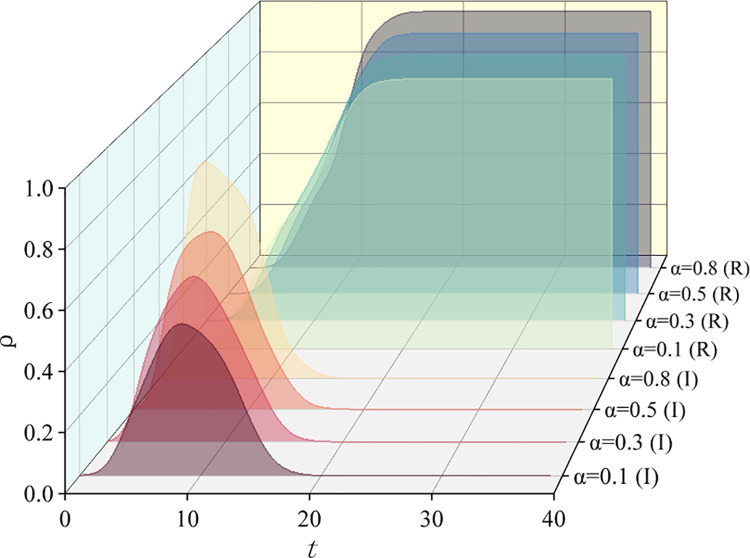
Effects of different transmission rates. Density change curves of I-state nodes and R-state nodes over time.

**Fig 9:**
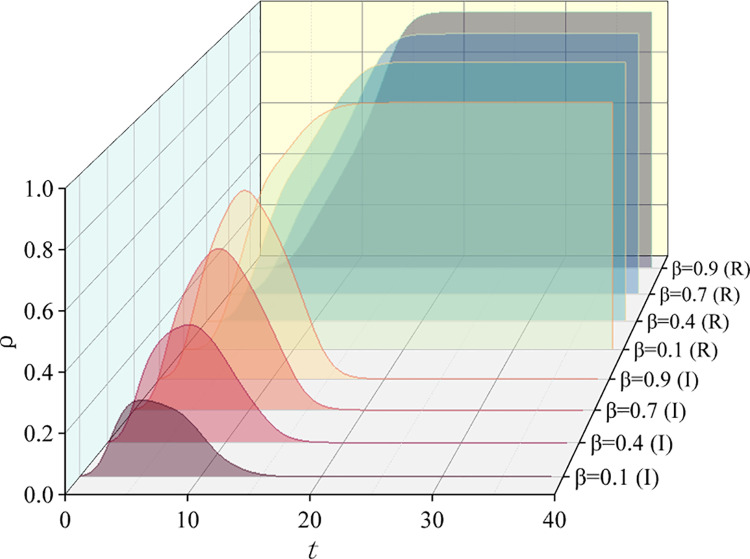
Effects of different transmission rates. Density change curves of I-state nodes and R-state nodes over time.

So, what impact do different propagation rates *α* and *β* have on information diffusion in hypernetworks? We set different values for *α*(0.1, 0.3, 0.5, 0.8) and *β*(0.1, 0.4, 0.7, 0.9) to observe the density changes of infected and removed state nodes. As shown in Fig 8 and Fig 9, as *α* and *β* increase, the peak of the infected state also increases, and the time for the removed state to reach steady-state decreases. During public opinion propagation, if the conversion rates *α* and *β* are high, more people will participate in information dissemination in a short period, creating a peak in the spread of public opinion. As a result, the time for the removed state to reach stability is also expedited.

### 4.3 Interaction dimensions and dissemination rates

**Fig 10:**
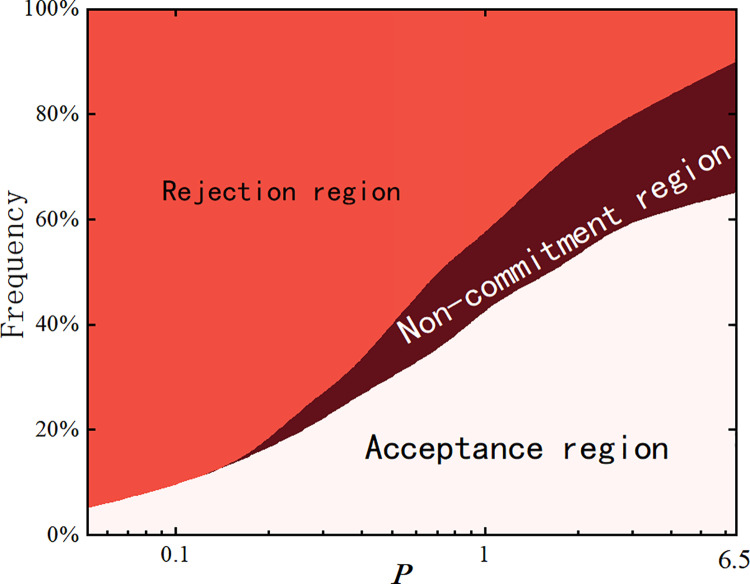
Schematic diagram of attitude dimension. The ratio of the rejection domain to the acceptance domain.

In this experiment, the study considers varying levels of participation as discussed in social judgment theory, and analyzes how different participation levels affect the standard deviation of users’ opinions by adjusting the range of interaction thresholds. When users have high participation, their rejection dimension is larger, the commitment dimension is nearly nonexistent, and the acceptance dimension is smaller. Conversely, with lower participation, the rejection dimension is smaller, while both the commitment and acceptance dimensions are larger. In the real world, people are susceptible to group influence and acceptance of majority opinions when faced with issues that do not involve their core personal interests. However, on critical issues, they may resist group pressure in order to protect their personal identity and beliefs. A diagram of opinion dimensions is shown in Fig 10, where *P* represents the ratio of the acceptance threshold to the rejection threshold.

Experimental parameter configuration: the initial opinions of all nodes obey a uniform random distribution from 0 to 1. Initially, one node is randomly selected for propagation, and the rest of the nodes are unknown states. Other parameters are set as follows:


N=5000,α=0.3,β=0.7,δ=0.7,λ=0.3,γ=0.3 (12)

**Fig 11:**
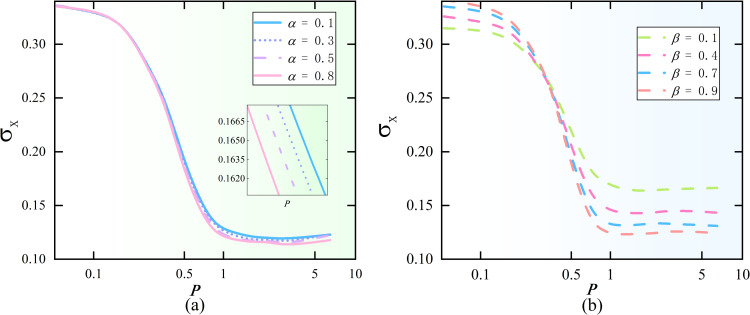
Distribution of standard deviation of steady-state view. Standard deviation under different attitudinal dimension ratios.

The results are shown in Fig 11(a) and (b). It can be observed that as the ratio *P* of the acceptance threshold $\varphi_{a}$to the rejection threshold $\varphi_{r}$increases, the standard deviation of the entire network decreases rapidly. This indicates that the communication and interaction radius among individuals increases, allowing for more opinions to be adopted, and most users’ opinions converge towards the mean *M*=0.5(±0.01). From the graph, it can be seen that the critical point occurs near $\varphi_{a}$=0.4, $\varphi_{r}$=0.45, and *P*=0.89. After the system reaches this critical point, the change in the standard deviation stabilizes, indicating that as interactions continue, individuals holding extreme opinions gradually decrease. Before the critical point, the standard deviation fluctuates significantly, reflecting the high diversity and considerable disagreement within the group. However, once this critical point is surpassed, the group’s opinion tends to converge, and the influence of extreme opinions weakens.

Based on simulation results under different values of *α* and *β*, the following analysis is made: α primarily increases the propagation speed, but its effect on the standard deviation is not significant. This is because the propagation speed mainly affects the efficiency and immediacy of information diffusion, but does not directly change the distribution or diversity of opinions in the network. In other words, when susceptibles quickly turn into infected nodes, this process reflects the rapid spread of information rather than the diversity of opinions or internal group disagreements. Therefore, even though the information propagation speed increases, the opinion divergence in the network may remain relatively stable, leading to no significant fluctuation in the standard deviation. On the other hand, increasing the conversion rate *β* from latent nodes to active propagators has a more pronounced effect on the standard deviation. This probability determines the manner and depth of information diffusion in the network. Latent nodes typically represent individuals who have been exposed to information but have not yet fully accepted or further disseminated it. When this conversion probability increases, more latent nodes become active propagators, thereby increasing the diversity and disagreement of opinions in the network. This process causes the opinion standard deviation to change, reflecting the increased complexity and diversity of opinions within the group.

**Fig 12:**
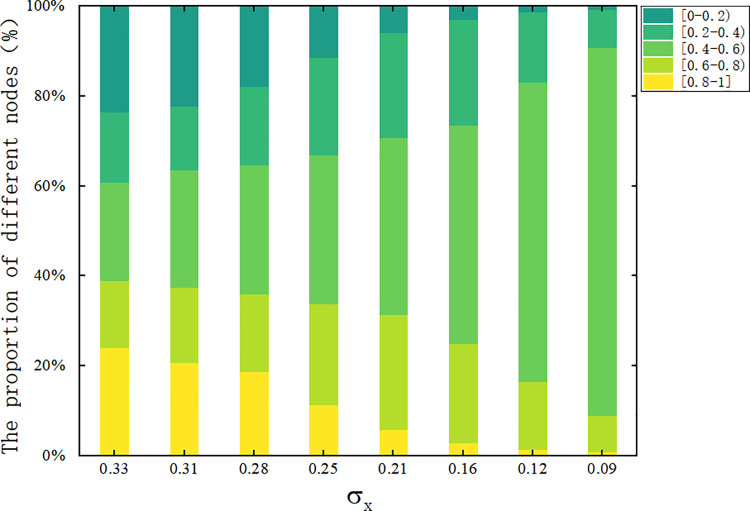
Proportion distribution of steady-state views. Distribution of final-state temporal opinions at different standard deviations.

Fig 12 gives the proportions of different standard deviation $\sigma_{x}$opinion distributions at steady state, and it can be seen that the proportions of extreme opinions are greater than 20% for $\sigma_{x}$=0.33 and 0.31, which indicates that users are more prone to the contrast rebound effect for larger values of the rejection domain. As $\varphi_{a}$increases, $\sigma_{x}$further decreases, and the proportion of extreme opinions gradually decreases to 0, and the opinions of the whole network approach to the range of [0.4–0.6]. Based on the above results, we will adopt the critical point $\varphi_{a}$= 0.4 and $\varphi_{r}$= 0.45 as the criteria for subsequent interactions.

### 4.4 Analysis of the effect of hyperedge size on opinion evolution

We now discuss the effect of hypergraph structure on viewpoint evolution. The internal structure of the network determines the paths of propagation and the objects of interaction, and we set up different average hyperdegrees of nodes and different average degrees of hyperedges to study their role.

Experimental parameter configuration: the initial opinions of all nodes obey a uniform random distribution from 0 to 1. Initially, one node is randomly selected for propagation, and the rest of the nodes are unknown states. Other parameters are set as follows:


N=5000,α=0.3,β=0.7,δ=0.7,λ=0.3,γ=0.3 (13)

**Fig 13:**
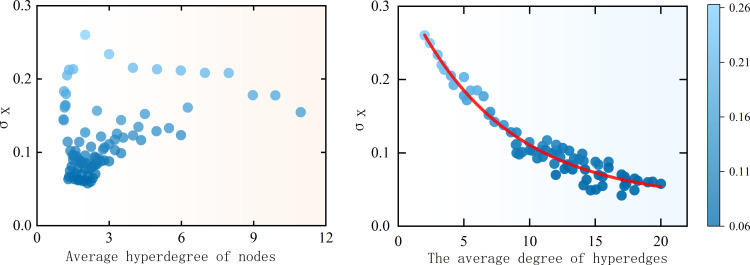
Average Exceedance (a) and average exceedance (b) of different nodes. (a) Distribution of standard deviation of nodes in a network with different sizes of mean supremacy (b) Distribution of standard deviation with different network mean supremacy degrees.

As shown in Fig 13(a), under different conditions of average node degree, a large amount of data clusters in the lower-left corner, and some nodes with higher average degrees do not show a very small$\sigma_{x}$. Furthermore, when the average node degree is in the range of 0–5, $\sigma_{x}$appears at nearly every value in the interval 0.06 to 0.3. This suggests that there is no significant or close relationship between the average node degree and the standard deviation. The following analysis can be made: when a user participates in multiple hyperedges, and the number of users in each hyperedge is small, even if the user’s average degree is high, it does not necessarily mean that they will easily adopt others’ opinions. This phenomenon also explains why, on diversified social platforms, although some users engage in multiple topic groups, they are not completely swayed by group consensus. This indicates that the diversity of opinions depends not only on the user’s social breadth but also on the structure and size of the groups they participate in.

To further verify this, we conducted another experiment where we statistically analyzed the average degree of hyperedges. The results, shown in Fig 13(b), reveal an interesting phenomenon: as the average degree of hyperedges increases, $\sigma_{x}$gradually decreases. After fitting these scatter points, we obtain a smooth curve. This trend confirms that, under interaction dimensions where the acceptance domain is not very small, opinion interactions in the hypernetwork mainly occur within hyperedges. As the number of nodes within a hyperedge increases, most users’ opinions converge towards the weighted sum mean, which results in a decrease in standard deviation. Therefore, compared to the average node degree, the average degree of hyperedges has a more significant impact on opinion interactions.

This trend is also insightful in daily life. For example, in families, friend groups, or work teams (similar to hyperedges), the larger the group size, the more likely individual opinions are to align with the group average under the influence of group pressure, leading to opinion convergence. In contrast, even if a person has a broad social network (i.e., a higher average node degree), if each of their social circles is relatively small and closed, the influence of these social relationships on their opinions is limited. They may still maintain independent opinions and not easily accept others’ views.

**Fig 14:**
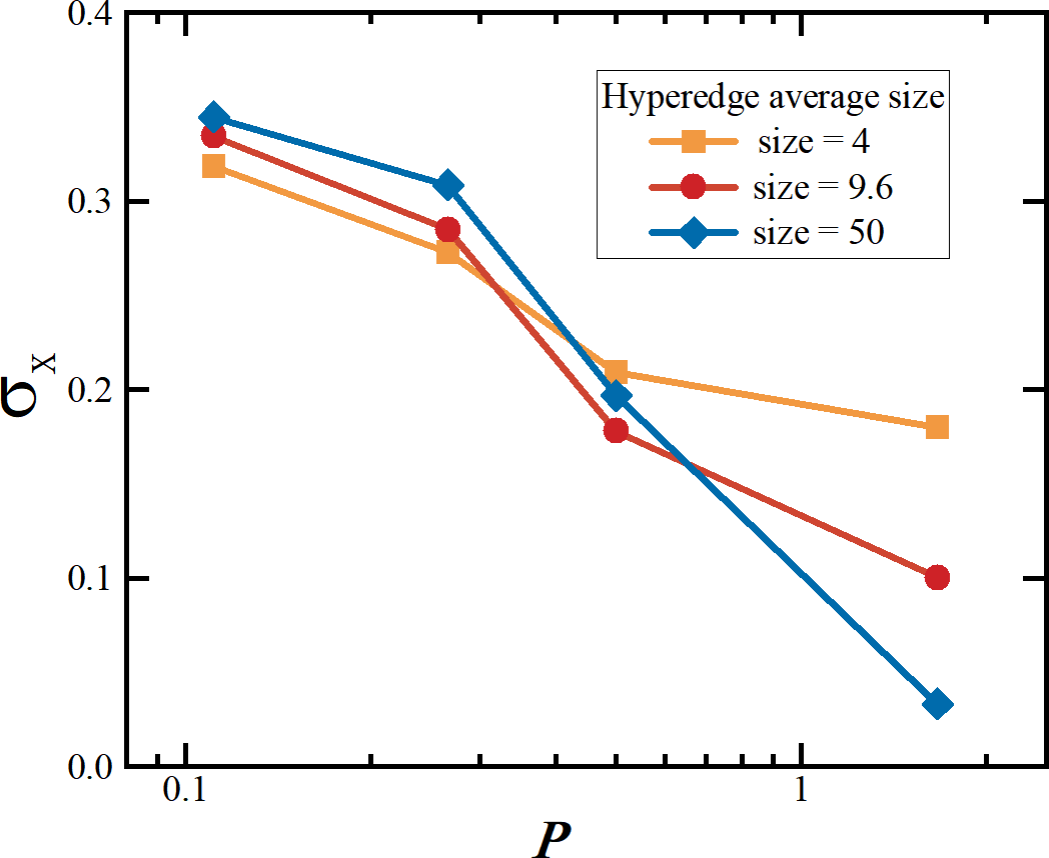
Results of different dimensions and different average hyperedge sizes. Distribution of standard deviations under different opinion interaction dimensions with different mean supermarginalities.

We then analyzed the impact of different interaction dimensions on the average hyperedge size. In related studies, prejudice and stereotyping have been considered as key factors such as social noise interference, prejudice assimilation and backfire effect, exploring the mechanisms by which individuals react when confronted with information that is inconsistent with their own opinions [47–49]. In this paper, it is considered as the case where the rejection domain is large. As shown in Fig 14, when the *P* is small, due to the larger rejection domain and lower acceptance domain, opinions in the network remain divergent, regardless of changes in the average size of the hyperedges. Moreover, the larger the average hyperedge, the more pronounced the contrast effect between opinions, resulting in greater disagreement. As the *P* increases, with the gradual expansion of the acceptance domain, the larger the average hyperedge, the more noticeable the opinion assimilation becomes, making it easier for the network to reach consensus. This suggests that, when the acceptance domain is not too small, increasing the size of the interaction group helps drive the convergence of opinions within the group.

An individual’s interaction dimension with different opinions is crucial for the formation of group consensus. When the acceptance threshold is low(Prejudice and stereotypes modeled as the “extended denial domain” phenomenon), even with a large group, opinion divergence may intensify. In large active groups on social media, people may refuse to adopt others’ opinions due to biases or entrenched positions (such as those related to politics, religion, ethnicity, gender equality, military issues, etc.), making it difficult for discussions to reach consensus. On the contrary, when the acceptance threshold is high, enlarging the group size helps facilitate consensus. In cross-departmental collaborations or large meetings (e.g., in scientific innovation, culture and art, cuisine, entertainment), open communication and listening can foster consensus on common issues. Therefore, merely increasing the number of participants without enhancing inclusiveness and openness may fail to effectively promote consensus, and may even backfire.

**Fig 15:**
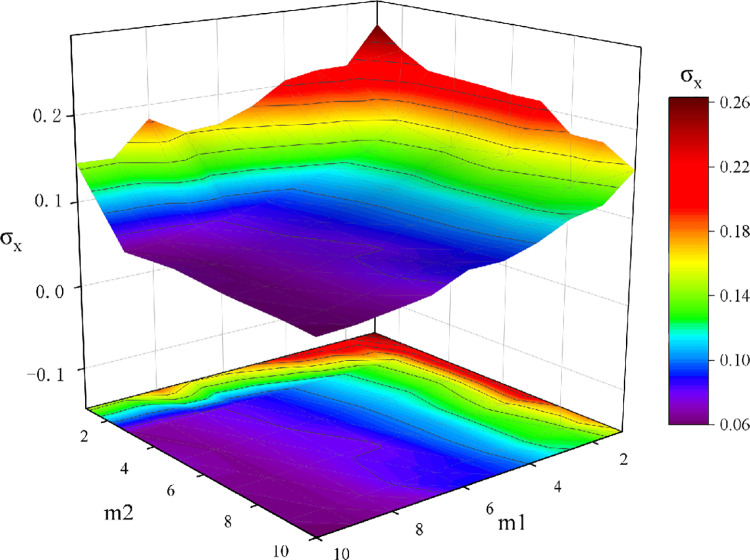
Standard deviation plot under different m_1_ and m_2_ generated hypergraphs. Distribution of end-state standard deviation under different new node m_1_ and old node m_2_ configurations in generating hypernetworks.

Finally, we analyzed the variable factors in the generation of the hypernetwork, specifically the number of new nodes *m*_1_ and old nodes *m*_2_ selected each time. In the previous network, these two parameters were randomly chosen within a certain range. We now fixed these parameters and analyzed the impact of different *m*_1_ and *m*_2_ combinations on$\sigma_{x}$. As shown in Fig 15, when both new and old nodes within the hyperedges are few, the standard deviation is higher. As *m*_1_ and *m*_2_ increase, the standard deviation gradually decreases. This also confirms the previous observation that the standard deviation $\sigma_{x}$decreases as the average hyperedge size increases.

This further supports the conclusion that, within a certain interaction dimension, the overall standard deviation of opinions in the network decreases as the average hyperedge size increases. This phenomenon closely mirrors real-world social and collaborative networks. When a group has fewer new and senior members, there tends to be greater disagreement within the group because the breadth and depth of communication are insufficient, making consensus difficult. This is similar to a newly formed project team, where differing opinions arise due to a lack of experience or mutual understanding among participants.

## 5 Conclusion and discussion

The interaction between information exchange and opinion evolution has long been a challenging issue in public opinion dynamics. To address this complex issue, this paper proposes an online social network opinion evolution model that incorporates higher-order interactions. Based on hypergraphs, this study considers the heterogeneity of online social network groups, constructs a scale-free hypergraph network topology, and introduces social judgment theory as the condition for user opinion interactions. The SEIR model is used as the framework for information dissemination, dynamically modeling the process of information spread and opinion evolution in online social networks. The paper investigates the high-order interaction evolution patterns in multi-node community structures, validates the model using real datasets, and further analyzes the impact of network topology, interaction dimensions, hyperedge size, and node degree on public opinion dynamics in online social networks. Our results indicate that group interactions significantly accelerate the process of reaching full consensus, aligning with recent research findings. For example, the study by N. Papanikolaou [50]. demonstrated that group interactions on hypergraphs facilitate more efficient information exchange through higher-order connections, thereby expediting consensus formation. Similarly, A. Hickok [51] highlighted how bounded confidence mechanisms on hypergraphs accelerate opinion convergence through group-level interactions. Additionally, L. Horstmeyer and C. Kuehn [52] demonstrated that higher-order interactions play a crucial role in shaping collective dynamics and facilitating consensus within a system.

Our study further uncovers the following key insights: 1. Compared to pairwise interaction networks, the group network topology built on hypergraphs has a higher propagation efficiency, and the opinion standard deviation of the network is lower at steady state. 2. The opinion interaction dimension is similar to the interaction domain in the HK or DW models. In high participation scenarios, the rejection domain is larger, and users are more likely to stick to their views, resulting in a divergent opinion state across the network. When participation is low, users’ acceptance increases, the radius for adopting opinions broadens, and the network reaches a convergent soft consensus. 3. At moderate interaction dimensions, we found that changes in the average node degree do not significantly affect consensus formation, whereas the average hyperedge degree has a significant effect. In a certain interaction dimension, as the average hyperedge degree increases, the opinion standard deviation in the network decreases, making consensus easier to achieve across the network.

Public opinion evolution in social networks is a particularly complex issue. Based on the above findings, we believe that in the current network environment, it is important to monitor the scope of information dissemination and the direction of user opinions to effectively guide public opinion. This research provides a theoretical and modeling foundation for the study of complex public opinion dynamics. However, there remains a gap between the model and real-world social networks. Future work should incorporate more real-life case studies to analyze and validate the current research, combining machine learning and hypergraphs to study public opinion trends.

## Supporting information

S1 File Enron e-mails hypergraph dataset. (TXT)

S2 File Hypergraph generation python code. (PY)
